# Evaluation of prescribing patterns in a German network of CAM physicians for the treatment of patients with hypertension: a prospective observational study

**DOI:** 10.1186/1471-2296-10-78

**Published:** 2009-12-10

**Authors:** Elke Jeschke, Thomas Ostermann, Horst C Vollmar, Matthias Kröz, Angelina Bockelbrink, Claudia M Witt, Stefan N Willich, Harald Matthes

**Affiliations:** 1 Havelhoehe Research Institute, Kladower Damm 221, 14089 Berlin, Germany; 2 Chair for Theory of Medicine, Integrative and Anthroposophic Medicine, University of Witten/Herdecke, Gerhard-Kienle-Weg 4, 58313 Herdecke, Germany; 3 Institute for General Practice and Family Medicine, University of Witten/Herdecke, Alfred-Herrhausen-Str 50, 58448 Witten, Germany; 4 Fraunhofer Institute for Systems and Innovation Transfer (ISI), Breslauer Str 48, 76139 Karlsruhe, Germany; 5 Institute for Social Medicine, Epidemiology and Health Economics, Charité University Medical Centre, 10117 Berlin, Germany

## Abstract

**Background:**

The management of hypertension is a key challenge in modern health systems. This study aimed to investigate hypertension treatment strategies among physicians specialized in complementary and alternative medicine (CAM) in Germany by analysing prescribing patterns and comparing these to the current treatment guidelines issued by the German Hypertension Society.

**Methods:**

In this prospective, multicentre observational study, which included 25 primary care physicians specialized in CAM treatment, prescriptions and diagnoses were analysed for each consecutive hypertensive patient using routine electronic data. Data analysis was performed using univariate statistical tests (Chi square test, Cochran-Armitage trend test). Multiple logistic regression was used to determine factors associated with antihypertensive medication.

**Results:**

In the year 2005, 1320 patients with 3278 prescriptions were included (mean age = 64.2 years (SD = 14.5), 63.5% women). Most patients were treated with conventional antihypertensive monotherapies (n = 838, 63.5%). Beta-blockers were the most commonly prescribed monotherapy (30.7%), followed by ACE inhibitors (24.0%). Combination treatment usually consisted of two antihypertensive drugs administered either as separate agents or as a coformulation. The most common combination was a diuretic plus an ACE inhibitor (31.2% of dual therapies). Patient gender, age, and comorbidities significantly influenced which treatment was prescribed. 187 patients (14.2%) received one or more CAM remedies, most of which were administered in addition to classic monotherapies (n = 104). Men (OR = 0.66; 95% CI: 0.54-0.80) and patients with diabetes (OR = 0.55; 95% CI: 0.42-0.0.73), hypercholesterolaemia (OR = 0.59; 95% CI: 0.47-0.75), obesity (OR = 0.74; 95% CI: 0.57-0.97), stroke (OR = 0.54; 95% CI: 0.40-0.74), or prior myocardial infarction (OR = 0.37; 95% CI: 0.17-0.81) were less likely to receive CAM treatment.

**Conclusions:**

The large majority of antihypertensive treatments prescribed by CAM physicians in the present study complied with the current German Hypertension Society treatment guidelines. Deviations from the guidelines were observed in one of every seven patients receiving some form of CAM treatment.

## Background

The management of hypertension is a key challenge in modern health systems. One of the most frequent chronic conditions and the most common treatable risk factor for cardiovascular disease, hypertension has been estimated to account for 6% of deaths worldwide [1] and for 26% of total mortality in Germany [2]. Hypertension was observed in 27% of women and 30% of men in Germany in a study by Thamm [3], and more recent studies have reported a prevalence of more than 50% in the general population [4].

Hypertension is the most common diagnosis made by general practitioners (GPs) in Germany [5], and antihypertensive agents are the second most frequently prescribed class of drugs, accounting for 15.4% of total drug expenditure [6]. Nevertheless, Germany is lagging behind internationally in areas such as hypertension awareness, treatment, and control [7]. Indeed, surveys have indicated that 41% of patients with known hypertension in this country are prescribed an inadequate dose of antihypertensive drugs or take these at dosing intervals that deviate from standard treatment recommendations [8]. Some experts have also criticized the use of newer and more expensive drugs, which in many cases may not be any more effective than older and less expensive treatments, such as diuretics [9].

The development, dissemination, and implementation of hypertension treatment guidelines are a key strategy for improving the care of hypertensive patients. Although several such guidelines have been published in Germany over the past decade, the most widely disseminated of these [10] were developed by the German Hypertension Society [11]. Moreover, recommendations have also been published for hypertensive patients themselves to ensure that they are kept abreast of the latest advances in treatment [12,13].

Some studies have indicated that complementary and alternative remedies from the areas of phytotherapy, homoeopathy, or anthroposophy have potential in the treatment of hypertension [14]. Whereas phytotherapy involves the use of undiluted plant extracts, homoeopathy relies on minerals, botanical substances, and other sources in diluted form. Anthroposophic remedies include preparations of botanical, mineral, or zoological origin, as well as chemical substances that are either undiluted or based on the homoeopathic principle of high dilution [15]. Three remedies in particular have shown some effects in the treatment of mild to moderate hypertension: crataegus [16,17], ginger [18], and Cardiodoron^® ^- a mixture of extracts from *Hyoscyamus niger *(Henbane) and the blossoms of *Primula veris *(Cowslip) and *Onopordum acanthium *(Scotch thistle) [19]. According to a systematic review by Ernst et al. [20], however, the effect sizes of complementary and alternative remedies in the treatment of hypertension are modest. To date, such remedies have not been included in evidence-based guidelines or patient recommendations for hypertension.

Nevertheless, the acceptance of complementary and alternative medicine (CAM) has grown over the past 20 years among physicians and patients alike. Patients with chronic diseases, such as hypertension, are increasingly seeking CAM treatment [21-23]. As a result, a growing number of physicians are being asked by their patients for advice on CAM or for referrals to its practitioners [24].

An important barrier to guideline adherence is the difficulty faced by physicians in reconciling patient preferences with guideline recommendations [25]. According to studies in a variety of primary care settings, patient expectations and preferences can influence the health care provided to them [26-28]. This issue may be particularly salient in the setting of CAM, as patients seeking treatment from physicians specialized in this field are likely to expect to receive some form of alternative therapy. The present study thus aims (a) to investigate adherence among primarycare physicians specialized in CAM to the hypertension treatment guidelines issued by the German Hypertension Society in 2005 and (b) to examine the use of complementary remedies among patients treated by these physicians by analysing prescribing patterns.

## Methods

In total, 25 primary care physicians in Germany participated in this prospective, multicentre observational study. All of them were members of the EvaMed Network, which aims to evaluate CAM remedies in usual care with regard to prescribing patterns, efficacy, and safety [29-31]. Physicians were recruited through the German National Association of Anthroposophic Physicians (*Gesellschaft Antroposophischer Ärzte in Deutschland*; GAÄD). A total of 362 physicians were contacted and informed about the EvaMed Network by standard mail and, in the event of non-response, four weeks later by telephone. For a physician to be eligible to participate in the study, his or her medical practice had to meet a number of technical requirements, including the presence of a special computerized patient documentation system (DocExpert, DocConcept, TurboMed, Duria, AdamedPlus, Medistar), a local area network (LAN) connection, and Microsoft Windows and Internet Explorer (i.e. as client software). A total of 38 physicians (10.5%) fulfilled the technical requirements, gave informed consent, and agreed to participate in the EvaMed network. Of these physicians, 13 specialized in paediatrics and dermatology were excluded from the study. Each of the remaining 25 physicians had practised for at least five years in primary care in addition to completing training in anthroposophic medicine.

The present study is based on secondary data provided by physicians. As such, the recommendations for good practice in secondary data analysis (e.g. anonymization of data on prescriptions and diagnoses) developed by the German Working Group on the Collection and Use of Secondary Data [32] were applied in full. In addition, the study was approved by the responsible data security official.

Data were included in the study if patients were at least 16 years old, had been diagnosed with hypertension, and had received pharmacological treatment for hypertension at least once during the study period (January-December 2005).

During the study, physicians continued to follow their routine documentation procedures, recording diagnoses and all prescriptions for each consecutive patient using their existing, computerized patient documentation system. These data were exported to the QuaDoSta postgreSQL database hosted in each practice [33]. Physicians used a browser-based interface to match individual diagnoses with the corresponding drugs or remedies that had been prescribed. Diagnoses were coded according to the 10th revision of the International Classification of Diseases (ICD-10). Prescribed drugs were documented using the German National Drug Code.

Study investigators identified all drugs and remedies prescribed for hypertension (i.e. ICD-10: I10 - I15). Each substance was classified using the Anatomical Therapeutic Chemical Index, and hypertensive drugs were clustered into classic antihypertensives (i.e. calcium channel blockers (CCBs), diuretics, beta-blockers, angiotensin converting enzyme (ACE) inhibitors, angiotensin II receptor antagonists, alpha-1 blockers, and antiadrenergic agents), and combination treatments (e.g. diuretics and beta-blockers, either as fixed-dose coformulations or as separate agents).

All statistical analyses were performed using SPSS 16.0 for Windows. Mean and standard deviations (SD) were calculated for continuous, normally distributed data. In cases where data were not normally distributed, medians and interquartile ranges (IQR) were reported. Subgroup analyses of prescribing rates were performed for patient age (under 40 years, 40-59 years, 60-79 years, 80 years and older), gender, and co-morbidities (e.g. diabetes mellitus, renal insufficiency, hypercholesterolaemia, coronary heart disease (CHD), post myocardial infarction, heart failure, stroke, obesity, asthma/chronic obstructive pulmonary disease (COPD)). The two-tailed Chi square test was used to analyse differences in prescription rates, and the Cochran-Armitage test was used as a measure of trend. A *P *value of less than 0.05 was regarded as indicating a statistically significant difference. Adjusted odds ratios (OR) and 95% confidence intervals (CI) were calculated using multiple logistic regression to determine factors associated with different hypertensive medications (CCBs, diuretics, beta-blockers, ACE inhibitors, angiotensin II antagonists, or CAM remedies).

## Results

### Physicians

Of the 25 participating physicians, 21 were GPs (84%) and 4 were internists (16%). The physicians did not differ significantly from the overall population of physicians certified in anthroposophy in Germany (n = 362) in terms of age (mean = 49.4; SD = 6.3 years vs. mean = 47.5; SD = 6.1 years; *P *= 0.709) or gender (60.0% vs. 62.2% men; *P *= 0.917), and were only slightly younger and consisted of a similar percentage of women compared to all office-based physicians in Germany (mean 52.0 years; 61.2% men) [34].

### Patients and prescriptions

During the study period, 8978 primary care patients (60.5% women) were treated by the participating physicians. Of the 3672 patients who were older than 16 years (40.9%), a total of 1320 (63.5% women) met the inclusion criteria (14.7%). The mean age of the included patients was 64.2 years (SD = 14.5). A total of 1613 comorbidities were recorded (median: 1; IQR [0, 2]). The highest comorbidity rates were seen in patients aged 60 to 79 years (1.20 in women and 1.61 in men) and patients aged 80 years and older (1.70 in women and 1.82 in men). In total, 32.4% of all patients had one, 19.8% had two, and 12.8% had three or more comorbidities. Thirty-five percent of patients (n = 462) had no reported comorbidities. Table 1 provides a detailed overview of comorbidities in participating patients.

**Table 1:** Sample of hypertensive patients subdivided according to comorbidities, age group, and gender

Age group [years]	Total	Without comorbidities	Comorbidities ^1^								
			
			Diabetes mellitus	Renal insufficiency	Hypercholesterolaemia	Coronary heart disease	Post myocardial infarcttion	Heart failure	Stroke	Obesity	Asthma/COPD
	**N**	**[n (%)]**	**[n (%)]**	**[n (%)]**	**[n (%)]**	**[n (%)]**	**[n (%)]**	**[n (%)]**	**[n (%)]**	**[n (%)]**	**[n (%)]**

Under 40	**23**	14 (60.9)	1 (4.3)	2 (8.7)	2 (8.7)	1 (4.3)	0 (0.0)	0 (0.0)	1 (4.3)	4 (17.4)	1 (4.3)
Female	**27**	17 (63.0)	1 (3.7)	2 (7.4)	2 (7.4)	2 (7.4)	0 (0.0)	0 (0.0)	0 (0.0)	6 (22.2)	4 (14.8)
Male											

40-59											
Female	**248**	124 (50.0)	23 (9.3)	8 (3.2)	62 (25.0)	12 (4.8)	0 (0.0)	2 (0.8)	9 (3.6)	62 (25.0)	10 (14.9)
Male	**164**	63 (38.4)	19 (11.6)	9 (5.5)	69 (42.1)	18 (11.0)	5 (3.0)	1 (0.6)	5 (3.0)	50 (30.5)	9 (19.6)

60-79											
Female	**406**	128 (31.5)	82 (20.2)	17 (4.2)	167 (41.1)	84 (20.7)	4 (1.0)	15 (3.7)	47 (11.6)	71 (17.5)	37 (55.2)
Male	**247**	71 (28.7)	64 (25.9)	18 (7.3)	96 (38.9)	68 (27.5)	17 (6.9)	10 (4.0)	24 (9.7)	30 (12,1)	24 (52.2)

80 or older											
Female	**161**	39 (24.2)	46 (28.6)	18 (11.2)	35 (21.7)	56 (34.8)	6 (3.7)	4 (2.5)	51 (31.7)	19 (11.8)	19 (28,4)
Male	**44**	6 (13.6)	10 (22.7)	7 (15.9)	13 (29.5)	17 (38.6)	3 (6.8)	2 (4.5)	17 (38.6)	5 (11.4)	9 (19,6)

**Total**	**1320**	**462 (35.0)**	**246 (16.6)**	**81 (6.1)**	**446 (33.8)**	**258 (19.5)**	**35 (2.7)**	**34 (2.6)**	**154 (11.7)**	**247 (18.7)**	**113 (8.6)**

^1 ^Double entries possible

During the study period, 1320 patients received a total of 3278 prescriptions (median prescriptions per patient and year: 1.5; IQR [1.0, 3.0]). Most patients (n = 838; 63.5%) received classic antihypertensive monotherapy, which accounted for 1705 prescriptions (52.0% of all prescriptions). A total of 482 patients (36.5%) received combination treatment with two antihypertensive drugs, which accounted for 845 prescriptions (25.8% of all prescriptions). Triple combinations were prescribed for 69 patients (5.2%), accounting for 104 prescriptions (3.2% of all prescriptions).

### Pharmacological therapy

A total of 187 patients (14.2%) were prescribed CAM remedies, accounting for 624 prescriptions (19.1% of all prescriptions). Of these prescriptions, 155 (4.7% of all prescriptions) were for CAM remedies administered as an adjunct to conventional therapy. Most frequently, this involved CAM prescribed in addition to classic monotherapy (n = 104), especially among women (n = 79). In total, 97 patients (7.3%) received antihypertensive CAM treatment exclusively (i.e. without any form of conventional therapy).

The most commonly prescribed agents were the beta-blocker metoprolol (9.5%), the ACE inhibitor enalapril (5.8%), the beta-blocker bisoprolol (4.4%), the CCB amlodipine (3.9%), the ACE inhibitor ramipril (3.3%), and the diuretic thiazide (3.0%). The most commonly prescribed CAM remedies were Cardiodoron^® ^(4.0%) and various aurum preparations (3.8%; e.g. Aurum/Belladonna comp^®^, Aurum metallicum praeparatum^®^, Aurum naturale D10/Punus spinosa, Summitates D5^®^).

For monotherapy, the most frequently prescribed agents were beta-blockers (30.7%), followed by ACE inhibitors (24.0%), CCBs (16.7%), and diuretics (15.7%). No differences could be seen in the prescription rates between the genders for specific monotherapies, such as beta-blockers (29.0% in men and 31.7% in women; *P *= 0.525) and angiotensin II receptor antagonists (14.6% in men and 10.1% in women; *P *= 0.332). The overall proportion of monotherapies also did not differ between men and women (*P *= 0.079; see table 2 for details). However, there were differences in the prescription rates for monotherapeutic agents between the various age groups. Beta-blockers, for example, were prescribed most frequently in patients aged less than 60 years, with 47.6% of patients aged between 20 and 39 years and 39.5% of patients aged between 40 and 59 years receiving these agents. In contrast, beta-blocker monotherapy was prescribed to only 28.1% of patients aged 60 to 79 years and 21.9% of patients aged 80 years and older (*P *for trend < 0.001). Among patients aged 80 years and older, diuretics (24.3%), ACE inhibitors (24.3%), CCBs (23.7%), and beta-blockers (21.9%) were prescribed with almost equal frequency. Compared to the average, however, these patients received a significantly higher proportion of CCBs and diuretics. Only every eighth patient (12.9%) received other forms of antihypertensive monotherapy (i.e. angiotensin II receptor antagonists (11.7%), alpha-1 blockers (0.2%), and antiadrenergic agents (1.0%)).

**Table 2:** Prescription of monotherapies according to age group and gender

Antihypertensives	Total	Gender		Age group [years]			
		
		Male	Female	Under 40	40-59	60-79	80 or older
	**[N (%)]**	**[n (%)]**	**[n (%)]**	**[n (%)]**	**[n (%)]**	**[n (%)]**	**[n (%)]**

CCBs^1^	**285 (16.7)**	101 (16.2)	184 (17.0)	12 (19.0)	47 (9.1)	148 (18.5)	78 (23.7)
- with CAM^2^	**9**	2	7	-	1	6	2

Diuretics	**267 (15.7)**	88 (14.1)	179 (16.6)	9 (14.3)	71 (13.7)	107 (13.4)	80 (24.3)
- with CAM	**28**	8	20	1	16	9	2

Beta-blockers	**523 (30.7)**	181 (29.0)	342 (31.7)	23 (36.5)	204 (39.5)	224 (28.1)	72 (21.9)
- with CAM	**27**	7	20	1	8	15	3

ACE inhibitors	**409 (24.0)**	158 (25.3)	251 (23.2)	17 (27.0)	106 (20.5)	206 (25.9)	80 (24.3)
- with CAM	**23**	3	20	1	6	10	6

Angiotensin II antagonists	**200 (11.7)**	91 (14.6)	109 (10.1)	1 (1.6)	79 (15.3)	104 (13.1)	16 (4.9)
- with CAM	**17**	5	12	-	7	8	2

Alpha-1 blockers	**4 (0.2)**	2 (0.3)	2 (0.2)	-	3 (0.6)	1 (0.1)	-
- with CAM	**0**	-	-	-	-	-	-

Antiadrenergic agents	**17 (1.0)**	4 (0.6)	13 (1.2)	1 (1.6)	7 (1.4)	6 (0.8)	3 (0.9)
- with CAM	**-**	-	-	-	-	-	-

**Total**	**1705 (100)**	**625 (100)**	**1080 (100)**	**63 (100)**	**517 (100)**	**796 (100)**	**329 (100)**

^1 ^Calcium channel blockers, ^1 ^complementary and alternative remedies

With regard to combination drug treatments (n = 845 prescriptions), the most commonly prescribed option was a diuretic plus an ACE inhibitor (31.2% of all dual therapies), followed by a diuretic plus an angiotensin II receptor antagonist (25.8% of all dual therapies). The latter option was prescribed significantly more often for women (29.1% in women vs. 19.4% in men; *P *= 0.018). Other dual and triple therapies ranged far behind. The most commonly prescribed fixed-dose coformulations were a diuretic plus an ACE inhibitor and a diuretic plus an angiotensin II receptor antagonist (table 3).

**Table 3:** Prescription of combination therapy according to age group and gender

Antihypertensives	Total	Gender		Age group [years]			
		
		Male	Female	Under 40	40-59	60-79	80 or older
	**[N (%)]**	**[n (%)]**	**[n (%)]**	**[n (%)]**	**[n (%)]**	**[n (%)]**	**[n (%)]**

**2-drug combinations**	**845 (100)**	**288 (100)**	**557 (100)**	**29 (100)**	**192 (100)**	**470 (100)**	**154 (100)**

Diuretic *plus*							

Beta-blocker							
- Fixed-dose coformulation	**67 (7.9)**	12 (4.1)	55 (9.9)	5 (17.2)	19 (9.9)	33 (7.0)	10 (6.5)
- Monopreparation	**53 (6.3)**	15 (5.2)	38 (6.8)	1 (3.4)	19 (9.9)	24 (5.1)	9 (5.8)

CCB^1^							
- Fixed-dose coformulation	**1 (0.1)**	1 (0.3)	-	-	-	1 (0.2)	-
- Monopreparation	**30 (3.6)**	11 (3.8)	19 (3.4)	-	3 (1.6)	15 (3.2)	12 (7.8)

ACE inhibitor							
- Fixed-dose coformulation	**208 (24.6)**	72 (25.0)	136 (24.4)	1 (3.4)	41 (21.4)	128 (27.2)	38 (24.7)
- Monopreparation	**56 (6.6)**	23 (8.0)	33 (5.9)	-	9 (4.7)	32 (6.8)	15 (9.7)

Angiotensin II antagonist							
- Fixed-dose coformulation	**207 (24.5)**	55 (19.1)	152 (27.3)	2 (6.9)	45 (23.4)	126 (26.8)	34 (22.1)
- Monopreparation	**11 (1.3)**	1 (0.3)	10 (1.8)	-	2 (1.0)	5 (1.1)	4 (2.6)

CCBs *plus*							

Beta-blocker							
- Fixed-dose coformulation	**-**	-	-	-	-	-	-
- Monopreparation	**38 (4.5)**	13 (4.5)	25 (4.5)	10 (34.5)	4 (2.1)	20 (4.3)	4 (2.6)

ACE inhibitor							
- Fixed-dose coformulation	**1 (0.1)**	1 (0.3)	-	-	-	1 (0.2)	-
- Monopreparation	**30 (3.6)**	11 (3.8)	19 (3.4)	1 (3.4)	9 (4.7)	16 (3.4)	4 (2.6)

Angiotensin II antagonist							
- Fixed-dose coformulation	**-**	-	-	-	-	-	-
- Monopreparation	**20 (2.4)**	9 (3.1)	11 (2.0)	-	4 (2.1)	14 (3.0)	2 (1.3)

Beta-blockers *plus*							
Other antihypertensive							
- Fixed-dose coformulation	**5 (0.6)**	-	5 (0.9)	-	-	4 (0.9)	1 (6.0)
- Monopreparation	**81 (9.6)**	52 (18.1)	29 (5.2)	7 (24.1)	33 (17.2)	33 (7.0)	8 (5.2)

Other	**37 (4.4)**	12 (4.2)	25 (4.5)	2 (6.9)	4 (2.1)	18 (3.8)	13 (8.4)

**3-drug combinations**	**104 (100)**	**58 (100)**	**46 (100)**	**3 (100)**	**31 (100)**	**58 (100)**	**12 (10)**

Diuretic *plus *ACE inhibitor *plus *CCB							
- Monopreparation							
	**2 (1.9)**	2 (3.4)	-	-	-	1 (1.7)	1 (8.3)

Diuretic *plus *beta-blocker *plus *^2^							
- Monopreparation	**57 (54.8)**	27 (46.6)	30 (65.2)	1 (33.3)	20 (64.5)	30 (51.7)	6 (50.0)

Diuretic *plus *antiadrenergic agents *plus *^2^							
- Monopreparation							
	**6 (5.8)**	5 (8.6)	1 (2.2)	-	4 (12.9)	2 (3.4)	-

Other	**39 (37.5)**	24 (41.4)	15 (32.6)	2 (66.7)	7 (22.6)	25 (43.1)	5 (41.7)

^1 ^Calcium channel blocker, ^2 ^CCB, ACE inhibitor, angiotensin II antagonist, alpha-blocker, or dihydrazine

Prescription rates for patients with specific comorbidities were also compared to overall prescription rates. Patients with heart failure were more likely to be prescribed ACE inhibitors (32.8% versus 24.5% for all patients) and diuretics (23.4% versus 15.7%), and less likely to be prescribed beta-blockers (12.5% versus 29.4%) (table 4). Patients with renal insufficiency were also less likely to be prescribed beta-blockers (15.7%), as were patients who had experienced a myocardial infarction (18.3%); these patients were more likely, however, to be prescribed a diuretic (21.3% in patients with renal insufficiency and 23.3% in patients after myocardial infarction).

**Table 4:** Prescription of monotherapies for comorbidities

Antihypertensives	Total	Without comorbidities	Comorbidities^3^								
			
			Diabetes mellitus	Renal insufficiency	Hypercholesterolaemia	CHD	Post myocardial infarction	Heart failure	Stroke	Obesity	Asthma/COPD
	**[N (%)]**	**[n (%)]**	**[n (%)]**	**[n (%)]**	**[n (%)]**	**[n (%)]**	**[n (%)]**	**[n (%)]**	**[n (%)]**	**[n (%)]**	**[n (%)]**

CCB^1^	**527 (17.1)**	88 (18.6)	55 (15.9)	26 (20.5)	97 (15.4)	58 (14.5)	12 (20.0)	12 (18.8)	56 (20.4)	47 (13.6)	76 (20.8)
- with CAM^2^	**14**	5	2	-	1	1	-	-	2	-	3

Diuretic	**514 (16.7)**	77 (16.2)	46 (13.3)	27 (21.3)	58 (9.2)	73 (18.3)	14 (23.3)	15 (23.4)	54 (19.6)	63 (18.2)	87 (23.8)
- with CAM	**41**	19	2	1	4	3	-	-	2	5	5

Beta-blocker	**882 (28.7)**	151 (31.9)	113 (32.8)	20 (15.7)	225 (35.7)	114 (28.5)	11 (18.3)	8 (12.5)	61 (22.2)	96 (27.7)	83 (22.7)
- with CAM	**36**	13	3	-	8	3	-	-	4	2	3

ACE inhibitor	**729 (23.6)**	94 (19.8)	75 (21.7)	37 (29.1)	147 (23.3)	113 (28.3)	17 (28.3)	21 (32.8)	79 (28.7)	85 (24.6)	61 (16.7)
- with CAM	**33**	6	4	3	6	9	-	-	2	2	1

Angiotensin II antagonist	**363 (11.8)**	64 (13.5)	50 (14.5)	11 (8.7)	86 (13.6)	38 (9.5)	5 (8.3)	7 (10.9)	18 (6.5)	48 (13.9)	36 (9.8)
- with CAM	**23**	8	1	1	4	1	-	-	1	4	3

Alpha-1 blocker	**20 (0.7)**	-	1 (0.3)	1 (0.8)	4 (0.6)	1 (0.3)	1 (1.7)	-	3 (1.1)	3 (0.9)	6 (1.6)
- with CAM	**-**	-	-	-	-	-	-	-	-	-	-

Antiadrenergic agent	**44 (1.4)**	-	5 (1.4)	5 (3.9)	14 (2.2)	3 (0.8)	-	1 (1.6)	4 (1.5)	4 (1.2)	8 (2.2)
- with CAM	**1**	-	-	-	-	-	-	-	-	-	1

**Total**	**3088 (100)**	**474 (100)**	**345 (100)**	**127 (100)**	**631 (100)**	**400 (100)**	**60 (100)**	**64 (100)**	**275 (100)**	**346 (100)**	**366 (100)**

^1 ^Calcium channel blocker, ^2 ^complementary remedy, ^3 ^double entries possible

With regard to combination treatments, patients with heart failure were more likely to be prescribed an ACE inhibitor plus a diuretic (60% versus 31.3% for all patients), as were patients with renal insufficiency (40.7%). Patients with diabetes were more likely to receive a diuretic plus an angiotensin II receptor antagonist than an ACE inhibitor plus a diuretic (28.4% versus 25.1%; see table 5).

**Table 5:** Prescription of combination therapy for comorbidities

Antihypertensives	Total	Without comorbidities	Comorbidities^3^								
			
			Diabetes mellitus	Renal insufficiency	Hypercholesterolaemia	CHD	Post myocardial infarction	Heart failure	Stroke	Obesity	Asthma/COPD
	**[N (%)]**	**[n (%)]**	**[n (%)]**	**[n (%)]**	**[n (%)]**	**[n (%)]**	**[n (%)]**	**[n (%)]**	**[n (%)]**	**[n (%)]**	**[n (%)]**

**2-drug combinations**	**1613 (100)**	**186 (100)**	**215 (100)**	**86 (100)**	**358 (100)**	**247 (100)**	**36 (100)**	**30 (100)**	**139 (100)**	**218 (100)**	**98 (100)**

**Diuretic *plus***											

Beta-blocker											
Fixed-dose coformulation	**103 (6.4)**	17 (9.2)	11 (5.1)	2 (2.3)	33 (9.2)	14 (5.7)	-	1 (3.3)	4 (2.9)	20 (9.2)	1 (1.0)
Monopreparation	**106 (6.6)**	14 (7.5)	12 (5.6)	5 (5.8)	20 (5.6)	15 (6.1)	2 (5.6)	-	9 (6.5)	16 (7.3)	13 (13.3)

CCB^1^											
Fixed-dose coformulation	**2 (0.1)**	-	-	-	-	1 (0.4)	-	-	1 (0.6)	-	-
Monopreparation	**58 (3.6)**	7 (3.8)	2 (0.9)	6 (7.0)	8 (2.2)	9 (3.6)	2 (5.6)	3 (10.0)	5 (3.2)	6 (2.8)	10 (10.2)

ACE inhibitor											
Fixed-dose coformulation	**372 (23.1)**	43 (23.1)	54 (25.1)	28 (32.6)	75 (20.9)	52 (21.1)	5 (13.9)	16 (53.3)	36 (26.1)	50 (22.9)	13 (13.3)
Monopreparation	**132 (8.2)**	4 (2.2)	15 (7.0)	7 (8.1)	24 (6.7)	26 (10.5)	5 (13.9)	2 (6.7)	11 (8.0)	21 (9.6)	17 (17.3)

Angiotensin II antagonist											
Fixed-dose coformulation	**396 (24.6)**	52 (28.0)	61 (28.4)	15 (17.4)	91 (25.4)	49 (19.8)	4 (11.1)	5 (16.7)	31 (22.5)	59 (27.1)	29 (29.6)
Monopreparation	**23 (1.4)**	2 (1.1)	2 (0.9)	2 (2.3)	7 (2.0)	4 (1.6)	-	1 (3.3)	3 (2.2)	1 (0.5)	1 (1.0)

**CCB *plus***											

Beta-blocker											
Fixed-dose coformulation	**-**	-	-	-	-	-	-	-	-	-	-
Monopreparation	**77 (4.8)**	15 (8.1)	12 (5.6)	5 (5.8)	11 (3.1)	11 (4.5)	4 (11.1)	-	8 (5.8)	7 (3.2)	4 (4.1)

ACE inhibitor											
Fixed-dose coformulation	**1 (0.1)**	1 (0.5)	-	-	-	-	-	-	-	-	-
Monopreparation	**65 (4.0)**	7 (3.8)	8 (3.7)	6 (7.0)	13 (3.6)	13 (5.3)	3 (8.3)	-	4 (2.9)	4 (1.8)	7 (7.1)

Angiotensin II antagonist											
Fixed-dose coformulation	**-**	-	-	-	-	-	-	-	-	-	-
Monopreparation	**35 (2.2)**	7 (3.8)	2 (0.9)	-	8 (2.2)	6 (2.4)	1 (2.8)	-	5 (3.6)	5 (2.3)	1 (1.0)

**Beta-blocker *plus***											
other antihypertensive											
Fixed-dose coformulation	**9 (0.6)**	1 (0.5)	-	-	4 (1.1)	1 (0.4)	-	1 (3.3)	2 (1.3)	-	-
Monopreparation	**172 (10.7)**	13 (7.0)	26 (12.1)	4 (4.7)	47 (13.1)	37 (14.9)	10 (27.8)	1 (3.3)	13 (9.4)	19 (8.7)	2 (2.0)

**Other**	**62 (3.8)**	3 (1.6)	10 (4.7)	6 (7.0)	17 (4.7)	9 (3.6)	-	-	7 (5.1)	10 (4.6)	-

**3-drug combinations**	**222 (100)**	**15 (100)**	**42 (100)**	**17 (100)**	**52 (100)**	**32 (100)**	**2 (100)**	**1 (100)**	**15 (100)**	**25 (100)**	**21 (100)**

Diuretic *plus *ACE inhibitor *plus *CCB											
Monopreparation											
	**8 (3.6)**	-	1 (2.4)	1 (5.9)	2 (3.8)	2 (6.3)	-	-	-	-	2 (9.5)

Diuretic *plus *beta-blocker *plus *^2^											
Monopreparation											
	**120 (54.1)**	6 (40.0)	26 (61.9)	5 (29.4)	26 (50.0)	19 (59.4)	1 (50.0)	-	9 (60.0)	17 (68.0)	11 (52.4)

Diuretic *plus *antiadrenergic agent *plu*s^2^											
Monopreparation											
	**10 (4.5)**	2 (13.3)	-	2 (11.8)	3 (5.8)	1 (3.1)	-	-	-	-	2 (9.5)

Other	**84 (37.8)**	7 (46.7)	15 (35.7)	9 (52.9)	21 (40.4)	10 (31.3)	1 (50.0)	1 (100)	6 (40.0)	8 (32.0)	6 (28.6)

^1 ^Calcium channel blocker, ^2 ^CCB, ACE inhibitor, angiotensin II antagonist, alpha-blocker, or dihydrazine, ^3 ^double entries possible

### Factors associated with specific hypertensive agents

Table 6 shows the adjusted OR for factors associated with specific antihypertensive agents independent of treatment regimen. Patient gender, age, and comorbidities had an impact on which treatment was prescribed. Men were more likely to receive an ACE inhibitor (OR = 1.44; 95% CI 1.20-1.74) or an angiotensin II receptor antagonist (OR = 1.62; 95% CI 1.26-2.09), but were less likely to receive a CAM remedy (OR = 0.66; 95% CI 0.54-0.80). Patients older than 60 were more likely to be prescribed a CCB (OR = 1.70; 95% CI 1.34-2.14), whereas patients under 60 were more likely to be prescribed a beta-blocker (OR = 0.50; 95% CI 0.41-0.60). With regard to specific comorbidities, patients with diabetes, for example, were more likely to be prescribed a beta-blocker (OR = 1.63; 95% CI 1.33-2.00) or an angiotensin II receptor antagonist (OR = 1.92; 95% CI 1.42-2.60). Concomitant diseases, such as diabetes mellitus (OR = 0.55; 95% CI 0.42-0.73), hypercholesterolaemia (OR = 0.59; 95% CI 0.47-0.75), obesity (OR = 0.74; 95% CI 0.57-0.97), stroke (OR = 0.54; 95% CI 0.40-0.74), and post myocardial infarction (OR = 0.37; 95% CI 0.17-0.81), reduced the odds of being prescribed a CAM remedy.

**Table 6:** Multiple logistic regression: Factors associated with special hypertensive medication

	CCBs	Diuretics	Beta-blockers	ACE inhibitors	Angiotensin II antagonists	CAM remedy
**Factor**	**Adjusted OR (95% CI)**					

Gender (male)	1.21 (0.99-1.48)	1.02 (0.83-1.25)	1.15 (0.96-1.36)	1.44 (1.20-1.74)*	1.62 (1.26-2.09)*	0.66 (0.54-0.80)*

Age (60 years or older)	1.70 (1.34-2.14)*	1.10 (0.88-1.38)	0.50 (0.41-0.60)*	0.87 (0.70-1.06)	0.75 (0.57-0.98)*	1.16 (0.95-1.42)

Comorbidies^1^	0.87 (0.64-1.18)	1.09 (0.79-1.49)	0.99 (0.76-1.30)	1.57 (1.16-2.11)*	0.46 (0.30-0.69)*	0.91 (0.69-1.21)

Diabetes	1.01 (0.79-1.29)	0.98 (0.76-1.25)	1.63 (1.33-2.00)*	0.85 (0.67-1.07)	1.92 (1.42-2.60)*	0.55 (0.42-0.73)*

Renal insufficiency	1.49 (1.09-2.05)*	1.33 (0.96-1.83)	0.58 (0.41-0.81)*	1.05 (0.76-1.44)	0.66 (0.40-1.10)	0.89 (0.62-1.28)

Hypercholesterolaemia	1.09 (0.87-1.37)	0.70 (0.56-0.88)*	1.29 (1.06-1.56)*	0.93 (0.76-1.15)	1.74 (1.28-2.36)*	0.59 (0.47-0.75)*
CHD	1.00 (0.79-1.26)	1.32 (1.05-1.66)*	1.68 (1.04-1.57)*	1.31 (1.05-1.62)*	1.12 (0.82-1.53)	0.82 (0.64-1.05)

Post myocardial infarction	1.27 (0.78-2.07)	1.43 (0.89-2.32)	1.30 (0.83-2.04)	1.69 (1.09-2.62)*	0.94 (0.47-1.89)	0.37 (0.17-0.81)*

Heart failure	0.72 (0.42-1.23)	0.98 (0.59-1.60)	0.30 (0.16-0.59)*	1.03 (0.64-1.64)	0.93 (0.44-1.97)	0.84 (0.51-1.38)

Stroke	1.22 (0.93-1.50)	1.18 (0.90-1.56)	1.02 (0.79-1.31)	1.22 (0.94-1.58)	1.29 (0.87-1.89)	0.54 (0.40-0.74)*

Obesity	0.98 (0.76-1.28)	1.49 (1.16-1.01)*	0.79 (0.64-0.99)*	1.11 (0.88-1.40)	1.07 (0.77-1.48)	0.74 (0.57-0.97)*

Asthma/COPD	1.41 (1.06-1.88)*	1.69 (1.23-2.23)*	0.94 (0.71-1.23)	0.73 (0.54-0.98)*	1.44 (0.97-2.13)	0.91 (0.67-1.24)

^1 ^at least one of the following indications in table 6

^* ^OR significantly different from 1

Power calculation was performed for the odds ratios from the logistic regression models in table 6 based on the algorithm provided in [35]. At a significance level of 0.05, the present study had 90% to 100% power to detect a change of 0.1 in the ORs (exceptions: post myocardial infarction: 0.42-0.68; heart failure 0.40-0.71).

## Discussion

A number of studies have investigated physician adherence to hypertension guidelines by analysing patient medical records or physician survey data. Although surveys are a useful tool, most have methodological limitations that could cause physician adherence to be underestimated. The present study relies, instead, on electronic prescription records and thus is not subject to the reporting or retrieval biases inherent to paper-based surveys.

The majority of patients in the present study were treated with classic antihypertensive monotherapies. Of these agents, beta-blockers were prescribed most frequently, followed by ACE inhibitors, CCBs, and diuretics. Patient gender, age, and the presence of comorbidities had a significant influence on which treatment was prescribed.

### Limitations

The present study has several important limitations. First, additional data on the hypertension diagnoses are lacking, such as blood pressure, smoking status, and lifestyle factors. Second, data on subsequent medication use in patients who switched physicians were unavailable, as were data on whether a patient was being treated for the first time with an antihypertensive agent. Third, from a statistical point of view, the subgroup analyses may be underpowered in cases where differences were small. Fourth, although our study population can be regarded as fairly representative of the overall population of German physicians specialized in anthroposophic treatment, it is not representative of all CAM physicians in Germany, who offer a range of further treatments, including those from the field of traditional Chinese medicine or integrative treatment programmes including Tai Chi [36,37]. Such treatments are not prescribed frequently, however. Finally, the present study lacks a direct comparison group. Further research on this subject would benefit from including a comparison group of CAM physicians, such as homoeopaths, or of conventional primary care physicians.

### Study population

Of the 3672 eligible patients who received a prescription during the study, 35.9% (n = 1320) were prescribed conventional and/or CAM treatment for hypertension at least once. This is comparable to other studies [3,4,38], in which a large proportion of patients have also been older (i.e. 65% aged 60 years or more) [39] and a higher CAM treatment rate was observed in women [40-43]. Although the types and rates of comorbidities in the present sample were generally similar to those observed in other surveys, the rates of diabetes and coronary heart disease experienced by our patients were slightly lower [44], which may be attributable to higher educational attainment or greater health awareness among users of complementary medicine [45].

### Prescription of antihypertensive treatments and adherence to guidelines

Each hypertensive patient received an average of 2.5 prescriptions per year, which at first glance might seem indicative of undertreatment. Indeed, this interpretation would be in accordance with the results of the HYDRA trial, in which patients were not provided with adequate hypertensive treatment by their GPs [38]. Nevertheless, the low average number of prescriptions per year in the present study may be attributable, at least in part, to two of the study's above mentioned limitations: (1) the inclusion in the cohort of patients being treated for the first time with antihypertensive therapy, and (2) the lack of data on subsequent medication use in patients who switched physicians.

In addition to lifestyle changes, current guidelines recommend initiating antihypertensive treatment using one or more agents from the following five drug classes: (thiazide) diuretics, beta-blockers, CCBs, ACE inhibitors, and angiotensin receptor blockers. Other substances are recommended only as adjunctive treatment. In the present study, only 2% of patients had conventional prescriptions that did not include any agents from these five classes. Monotherapies accounted for 64% of all prescribed treatments, and the proportion of patients receiving monotherapy was high compared to the German MONICA Study (50%) and similar to that observed in Israel (64%) and Finland (59%) [46]. In accordance with other studies, beta-blockers were prescribed most often, followed by ACE inhibitors and the combination of a diuretic plus an ACE inhibitor [39,46]. When available, fixed-dose coformulations were used frequently, accounting for 55.4% of all two-drug combinations; this is likely attributable to the desire to increase patient compliance.

The finding that there are significant differences in the prescription rates for various antihypertensive agents according to age, gender, and comorbidities is in accordance with the results reported by Pears et al. [47].

After being adjusted for comorbidity and age, the results of the present study show that women were less likely to receive ACE inhibitors or angiotensin II receptor antagonists [48,49]. Beta-blockers were prescribed primarily for patients under the age of 60. These findings concur with those in comparable national and international surveys of antihypertensive drug use in primary care [38].

In 2003 Pittrow et al. showed that in German primary care, hypertensive patients with comorbidities do not receive the individualized treatment recommended in the current Germany Hypertension Society guidelines. For example, although CCBs and beta-blockers are the preferred option in CHD patients with hypertension, ACE inhibitors and diuretics were prescribed most frequently [39]. In the present study, there was also a significantly higher prescription rate for diuretics (OR = 1.32; 95% CI 1.05-1.66) or ACE inhibitors (OR = 1.31; 95% CI 1.05-1.62) in CHD patients; however, the OR for the use of beta-blockers, which are recommended in the German Hypertension Society guidelines, was even higher (1.68; 95% CI 1.04-1.57). For patients with diabetes, the OR for receiving beta-blockers, which would also address the high cardiac risk associated with diabetes, was significantly greater than 1 (OR = 1.63; 95% CI 1.33-2.00) [39]. The OR for patients with prior myocardial infarction was significantly greater than 1 for ACE inhibitors (OR = 1.69; 95% CI 1.09-2.62), which are also recommended in the guidelines, but there was not a significantly lower prescription rate for CCBs (OR = 1.27; 95% CI 0.78-2.07), which are not recommended.

The prescription rates for CCBs in patients with heart failure (12 out of 527 patient, or 2.3%), beta-blockers in asthma/COPD patients (83 out of 882 patients, or 9.4%), and alpha-1-blockers in patients with renal insufficiency (1 out of 20 patients, or 5.0%) - all of which are contraindicated in the respective patient group - were below 10%. After adjustment for other factors, multiple logistic regression did not result in significantly lower prescription rates for CCBs in patients with heart failure (OR = 0.72; 95% CI 0.42-1.23) or for beta-blockers in patients with asthma/COPD (OR = 0.94; 95% CI 0.71-1.23).

### CAM remedies

Although CAM remedies are not recommended in any of the currently available guidelines, they were included in 19.1% of the prescriptions in the present study. This is not surprising considering that most patients who visit CAM physicians likely do so because they are seeking alternative treatments. It may be that this group of patients had only mild hypertension and received CAM remedies in addition to any conventional treatment and recommendations for lifestyle changes. The results of the present study strongly suggest that CAM remedies were prescribed mainly as adjuncts to antihypertensive monotherapy, most likely as a way to stimulate and harmonize the rhythmic system, assisting the body in regulating blood pressure with its own resources [50]. As expected, CAM medication was prescribed primarily to women and patients without comorbidities [40-43]. Altogether, the prescription rate for CAM remedies among patients with hypertension was very low compared to that among patients with other diseases, such as acute upper respiratory tract infections (79.8%), otitis media, or dorsopathies, in the same network of physicians [51].

### Elderly patients with hypertension

Due to methodological limitations, most earlier studies on hypertension have excluded patients older than 65, thus failing to evaluate a subgroup of patients whose importance will only increase in the coming decades. The Hyvet study published in 2008 [52] was therefore an important addition to the literature, demonstrating the benefits of treating hypertension in patients aged 80 years or older. In accordance with current German Hypertension Society guidelines, the results of the present study show higher prescription rates for CCBs and diuretics in elderly patients, as well as an almost equal distribution of classic antihypertensive monotherapies among patients aged 80 years and older.

## Conclusions

This paper is the first to provide insight into the hypertension treatment strategies of physicians specialized in CAM treatment using routine data from practice information systems. Despite the limitations of the available data, it can be concluded that the large majority of antihypertensive treatments prescribed by CAM physicians in the present study were compliant with the current guidelines of the German Hypertension Society. Deviations from these guidelines were seen in one of every seven patients receiving some form of CAM treatment.

## Competing interests

The authors declare that they have no competing interests.

## Authors' contributions

EJ helped design the study and acquire data, performed the statistical analysis, and drafted the manuscript. TO made substantial contributions to data interpretation and statistical analysis. HCV, AB, MK, and CMW helped interpret the data and draft or critically revise the manuscript. HM conceived of the study and participated in its design and coordination. MK, CMW, SNW, and HM gave final approval of the version to be published. All authors read and approved the final manuscript.

## Pre-publication history

The pre-publication history for this paper can be accessed here:

http://www.biomedcentral.com/1471-2296/10/78/prepub
